# Rheumatoid arthritis-relevant DNA methylation changes identified in ACPA-positive asymptomatic individuals using methylome capture sequencing

**DOI:** 10.1186/s13148-019-0699-9

**Published:** 2019-07-31

**Authors:** Xiaojian Shao, Marie Hudson, Ines Colmegna, Celia M. T. Greenwood, Marvin J. Fritzler, Philip Awadalla, Tomi Pastinen, Sasha Bernatsky

**Affiliations:** 1 Department of Human Genetics, McGill University, Montréal, Canada; 2 The McGill University and Génome Québec Innovation Centre, Montréal, Canada; 3 Department of Medicine, McGill University, 5252 Boul. de Maisonneuve Ouest, Rm 3F.51, Montréal, H4A 3S5 Canada; 4 Division of Rheumatology, Jewish General Hospital, Montréal, Canada; 5 Lady Davis Institute, Jewish General Hospital, Montréal, Canada; 6 Division of Rheumatology, McGill University, Montréal, Canada; 7 Cumming School of Medicine, University of Calgary, Calgary, Canada; 8 Ontario Institute for Cancer Research, Toronto, Canada; 9 Department of Molecular Genetics, University of Toronto, Toronto, Canada; 10 Center for Pediatric Genomic Medicine, Children’s Mercy, Kansas City, MO USA; 11 Current address: Digital Technologies Research Centre, National Research Council Canada, Ottawa, Ontario Canada

**Keywords:** Rheumatoid arthritis, Anti-citrullinated protein antibody positivity, DNA methylation, Targeted bisulfite sequencing, Differentially methylated regions

## Abstract

**Objective:**

To compare DNA methylation in subjects positive vs negative for anti-citrullinated protein antibodies (ACPA), a key serological marker of rheumatoid arthritis (RA) risk.

**Methods:**

With banked serum from a random subset (*N* = 3600) of a large general population cohort, we identified ACPA-positive samples and compared them to age- and sex-matched ACPA-negative controls. We used a custom-designed methylome panel to conduct targeted bisulfite sequencing of 5 million CpGs located in regulatory or hypomethylated regions of DNA from whole blood (red blood cell lysed). Using binomial regression models, we investigated the differentially methylated regions (DMRs) between ACPA-positive vs ACPA-negative subjects. An independent set of T cells from RA patients was used to “validate” the differentially methylated sites.

**Results:**

We measured DNA methylation in 137 subjects, of whom 63 were ACPA-positive, 66 were ACPA-negative, and 8 had self-reported RA. We identified 1303 DMRs of relevance, of which one third (402) had underlying genetic effects. These DMRs were enriched in intergenic CpG islands (CGI) and CGI shore regions. Furthermore, the genes associated with these DMRs were enriched in pathways related to Epstein-Barr virus infection and immune response. In addition, 80 (38%) of 208 RA-specific DMRs were replicated in T cells from RA samples.

**Conclusions:**

Sequencing-based high-resolution methylome mapping revealed biologically relevant DNA methylation changes in asymptomatic individuals positive for ACPA that overlap with those seen in RA. Pathway analyses suggested roles for viral infections, which may represent the effect of environmental triggers upstream of disease onset.

**Electronic supplementary material:**

The online version of this article (10.1186/s13148-019-0699-9) contains supplementary material, which is available to authorized users.

## Introduction

Rheumatoid arthritis (RA) is an autoimmune disease and the most common chronic inflammatory polyarthritis [1, 2] with a marked female predominance [3]. It is a complex disease triggered by the combination of risk alleles from different susceptibility genes and exposure to environmental factors. During a pre-clinical course lasting up to several years, RA-related antibodies such as anti-citrullinated peptide antibodies (ACPA) can be detected even prior to clinical manifestations as evidence of early immune dysregulation [4, 5]. Though links between environmental and genomic events are incompletely understood, environmental effects may be mediated through epigenetic mechanisms [6–8].

Altered DNA methylation patterns have been identified in clinical RA [9, 10]. Indeed, global hypomethylation was shown in T cells of RA patients [11–13] and hyper- and hypomethylation of specific genomic sites were also shown in synovial fibroblasts [14–16]. RA synovial fibroblasts display global DNA hypomethylation [17, 18], and more complicated patterns are seen in CD4+ T cells or peripheral blood mononuclear cells (PBMCs) [12, 19–21]. Prominent DNA methylation alterations mediating genetic risk in RA have been found in the major histocompatibility complex (MHC) region [6]. Comparing DNA methylation patterns in subjects with and without RA (including subjects with ACPA positivity but no clinical signs) would have implications for understanding causal pathways related between epigenetic abnormalities and disease. However, targeted array-based platforms such as Illumina Human MethylationHM450K (HM450K) preferentially cover CpG-dense regions, which are not necessarily relevant in autoimmune disease [22].

We compared ACPA-positive vs ACPA-negative asymptomatic subjects using custom methylC-capture sequencing (MCC-Seq) [22, 23], a next-generation sequencing capture approach. This comprehensive MCC-Seq panel encompasses ~ 5 million CpGs [22], representing regulatory regions in peripheral leukocytes as well as hypomethylated regions in whole blood. This allows study of potentially disease-associated CpGs in distal regulatory elements and eliminates direct interference by genetic variants. We identified novel ACPA/RA-associated CpGs and regions and replicated our findings in an independent RA sample. Moreover, we demonstrated DMR-associated genes enriched in pathways related to viral infections and immune response.

## Methods

### Patients

#### General population subjects

Our analyses were based within the CARTaGENE platform (https://cartagene.qc.ca/), made of 19,995 general population subjects (aged 40 to 69) across four census metropolitan areas: Montreal, Quebec City, Sherbrooke, and Saguenay-Lac**-**Saint**-**Jean, all in the province of Quebec, Canada. Participants were randomly selected from the provincial health insurance registries (fichier administratif des inscriptions des personnes assurees de la Regie de l’assurance maladie du Quebec, RAMQ). This excluded residents of First Nations reserves or long-term health care facilities or prisons. The participants were selected according to the age distribution in the four areas, to obtain a representative sample.

Using bio-banked sera from a random subset (*N* = 3600) of CARTaGENE subjects, we performed Inova enzyme-linked immunosorbent assay (Quanta Lyte, CCP3 IgG: Inova Diagnostics Inc., San Diego, CA) and identified 69 ACPA-positive subjects (1.9%). Among them, 18 were highly positive (ACPA > 60 optical density, OD units) and the rest were low or medium positive (20–60 OD), all but one had ACPA > 40 OD). ACPA-positive patients were matched for age, sex, and smoking status to ACPA-negative (ACPA ≤ 20 OD) CARTaGENE subjects (*N* = 68). Whole blood from ACPA-positive and ACPA-negative patients (*n* = 137) was used to extract DNA for the current analyses. Among the 137 subjects, 8 samples had self-reported RA, 6 of whom were ACPA-positive and 2 ACPA-negative. Proportions of circulating cell sub-types (standard cell count and differential), including monocyte, lymphocyte, neutrophil, eosinophil, and basophil, were available at the time of the sampling.

#### Rheumatoid arthritis patients

Nine new-onset (symptom duration < 1 year) treatment-naïve RA patients and 13 control subjects were used to validate the epigenome-wide association study (EWAS) analysis from the CARTaGENE cohort. These subjects were recruited from the Jewish General Hospital and McGill University Health Centre arthritis clinics. Forty milliliters of blood was collected from each subject, and CD4^+^ T cell-positive selection (anti-CD4 microbeads, MiltenyiBiotec, and auto-MACS) was performed. Samples with purity > 95% (using flow cytometry) were sequenced.

### Methylation sequencing

Methylation capture sequencing (MCC-Seq) was performed as previously described [22, 23]. DNA methylation of each CpG was measured by the number of methylated reads over the total number of sequenced reads. Details are provided in Additional file 1. This immune panel covers the majority of human gene promoters, methylation footprint regions [24] observed in blood, blood-cell-lineage-specific enhancer regions, CpGs from Illumina Human Methylation 450 Bead Chips, and published autoimmune-related SNPs as well as SNPs in their LD regions with *r*^2^ > 0.8. Overall, it covers 4,861,805 CpGs [22].

### Statistical analyses

To look for associations between DNA methylation and ACPA levels in CARTaGENE subjects, we built generalized linear regression models (GLM) using the methylation proportion inferred from the combination of methylated reads and unmethylated reads as a binomially distributed response variable, and ACPA status (e.g., positive or negative) as a predictor, with sex, age, and smoking status as covariates. Here we used the R function *glm()* and the binomial family to fit each model and calculated *p* values for variables of interest with Wald-type tests. Dose effects were considered in a similar model with ACPA status as an ordinal variable (ACPA negative, medium positive, and high positive as 0, 1, and 2, respectively). A third analysis compared CARTaGENE subjects with self-reported RA to the non-RA CARTaGENE subjects (ACPA-positive and ACPA-negative). All analyses were adjusted for blood cell-type composition, by adding the proportion of each blood cell subtype (i.e., monocyte, lymphocyte, neutrophil, eosinophil, and basophil) to the models as additional covariates. DNA methylation measures on the X and Y chromosomes from EWAS analysis were excluded. From the distribution of *p* values obtained for the autosomal CpGs, false discovery rate *q* values were estimated using the R package *q* values [25, 26]; *q* values less than 0.01 were considered significant. Non-variable CpGs (standard deviation = 0) were removed to reduce the multiple testing burden. For some CpGs, the number of individuals with sufficient sequencing coverage (≥ 15×) was low (e.g., < 30 samples); these CpGs were removed from our analyses, to minimize the impact of low measurement accuracy. To assess potential regional clustering of significant CpGs, we selected CpGs with differential methylation (DMCs) for ACPA-positive vs ACPA-negative CARTaGENE subjects and created a candidate region around these sites of up to 200 bp both upstream and downstream. Within these candidate regions, all consecutive CpGs with methylation changes in the same direction (with nominal *p* value < 0.01) were merged. Regions with at least 3 CpGs fulfilling these criteria were considered differentially methylated regions (DMRs, Additional file 2: Figure S1A). For the analysis of the CD4+ T cells of RA cases and controls, we fit a simpler binomial regression model without including additional covariates for smoking, cell types, or sex, due to the sample size available.

To investigate genetic effects on DNA methylation, methylation quantitative trait locus (meQTLs) analyses were performed (Additional file 2: Figure S1B). Genotypes were inferred directly from the MCC-Seq data using BisSNP [27] with an additional step fetching the genotype of homogenous reference alleles from the aligned BAM files. Bi-allelic SNPs were retained for analysis where SNPs had at least a read depth ≥ 10× and where more than 70% of the subjects had genotype calls. CpGs measured in more than 68 individuals (i.e., > 50% of all individuals) and with large variance (i.e., variance in the top 50% of all CpGs) were selected for this analysis. By considering possible SNP *cis*-effects within 250 kb of a CpG (i.e., a 500-kb window), meQTLs were calculated using MatrixEQTL with default parameters [28], correcting *p* values using the false discovery rate approach [29]. For genotype adjustment of the models, the genotypes of identified significant meQTLs (*q* value < 0.01) within the 500-kb window were added into the binomial regression models.

### Genome features and enrichment analysis

Genome feature files and annotation tables, including transcription start sites (TSSs), 3’UTRs, 5’UTRs, first exons, exons, introns, and transcription end sites (TESs), were downloaded from the UCSC genome browser version of hg19. The promoter regions were defined as TSS1500 (1500 bp from TSSs). CpG islands (CGI) were defined as per the UCSC genome browser. CGI shores were defined as the 2-kb flanking sequences on either side of CGIs; shelves were defined as the 2-kb flanking sequences beyond the shores. Genome feature enrichment analyses of RA-associated DMRs were performed using Fisher’s exact test for significance where the background set included all testable CpGs. The closest genes for DMRs were annotated using homer [30] (version 4.9.1). Pathway enrichment analyses were also performed using homer [30]. Gene sets detected from the immune panel were used as the background set.

## Results

### CARTaGENE subjects

Table 1 characterizes the sampled 137 CARTaGENE subjects included in this study (63 ACPA-positive and 66 ACPA-negative without self-reported RA, and 8 females with self-reported RA).

**Table 1:** CARTaGENE subjects: ACPA-positive, ACPA-negative, and RA

	All subjects (*N* = 137)	ACPA-positive (*N* = 63)	ACPA-negative (*N* = 66)	Self-reported RA (*N* = 8)
Mean age (range)	55.2 (40.4–69.9)	55.6 (40.4–69.9)	54.9 (41.7–69.2)	54.6 (45.4–69.8)
Female, *N* (%)	89 (64.9%)	39 (61.9%)	42 (63.6%)	8 (100%)
Smoker, *N* (%)				
Current	28 (20.5%)	14 (22.2%)	13 (19.7%)	1 (12.5%)
Past	58 (42.3%)	27 (42.9%)	27 (40.9%)	4 (50%)
Never	51 (37.2%)	22 (34.9%)	26 (39.4%)	3 (37.5%)

Average sequence genome coverage in targeted regions was 15×. Over 6 million CpGs captured in at least one sample and consisting of the targeted CpGs and flanking CpGs within 500 bp of the targeted panel, underwent downstream analysis. When restricting attention to CpGs with good coverage in at least 30 samples, 5 million CpGs remained for analysis. See Additional file 3: Figure S2 for details on read and sample coverage.

### Genome-wide analysis of DNA methylation in ACPA healthy and RA subjects

**Fig. 1:**
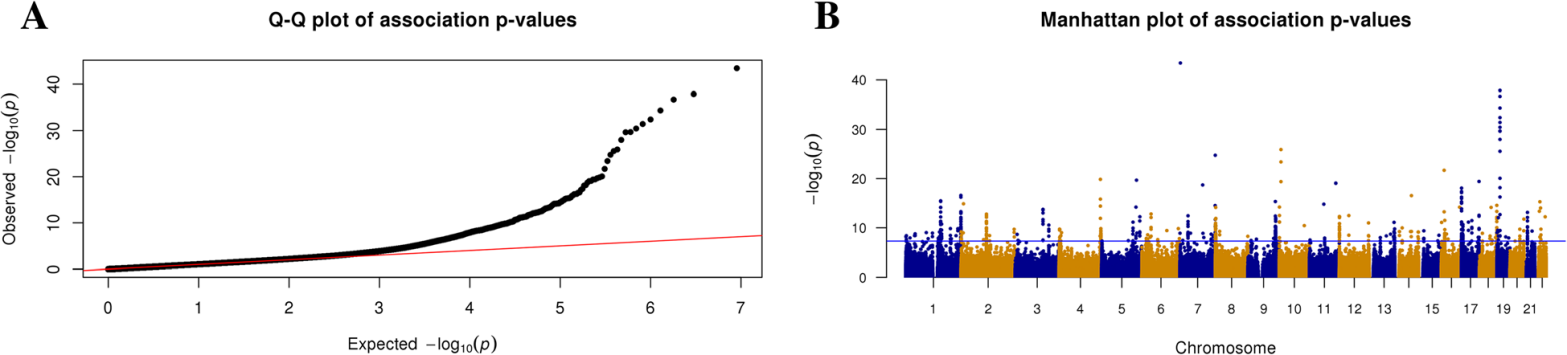
Genome-wide distribution of significant CpG sites from EWAS analysis. **a** qq-plot of the *p* values from the EWAS associations between ACPA-positive and ACPA-negative after adjustment for cell-type heterogeneity. Genomic control value lambda = 1.05 indicates no obvious inflation. The *x*-axis indicates the expected −log10 (*p* values), whereas the *y*-axis shows the observed −log10 (*p* values). **b** Manhattan plot of the *p* values from the EWAS associations. The *x*-axis indicates genomic locations of the CpGs, and the *y*-axis shows −log10 (*p* values) of the associations

In EWAS comparisons of ACPA-positive and ACPA-negative subjects (excluding the 8 self-reported RA patients), we identified 2047 DMCs (*q* value ≤ 0.01); 668 were hypomethylated and 1379 hypermethylated (model I, see Table 2 for a summary of all models fit and the number of DMCs and DMRs identified). After adjusting for blood cell heterogeneity (model II), 1295 (63.3%) of the methylation differences remained significant and 614 new DMCs were identified, leading to a final identification of 1909 DMCs (679 hypomethylated and 1230 hypermethylated). The genome-wide distribution of significant CpG sites for model II is shown in Fig. 1 and Additional file 5: Table S1. At a *q* value < 0.1 (from the cell composition adjusted model), 85.3% of the DMCs in model I remained significant in model II (Additional file 4: Figure S3). Unless stated specifically, all downstream models were based on cell-type adjusted results.

**Table 2:** Summary of differentially methylated CpGs from different models

Models	Phenotype groups	#CpG tested	#DMCs	#hypoDMCs	#hyperDMCs	#DMRs	#hypoDMRs	#hyperDMRs
I	ACPA-positive vs. ACPA-negative	4,733,057	2047	668	1379	623	210	413
II	ACPA positive vs. ACPA-negative with cell type adjustment	4,635,909	1909	679	1230	509	158	351
III	Self-reported RA vs. ACPA healthy with cell type adjustment	4,109,916	955	435	520	249	81	168
IV	ACPA ordinal (negative/medium/high) with cell type adjustment	4,049,218	4475	869^a^	761	1303	62^a^	60
V	T cell RA patient vs. controls	3,262,817	1595	868	727	502	202	300

^a^The number of hypo/hyper DMCs/DMRs were calculated based on the directional changes of DNA methylation across ACPA-negative, ACPA-medium-positive, and ACPA-high-positive

After performing regional clustering of significant DMCs from model II, we defined 509 DMRs when comparing ACPA-positive vs. ACPA-negative subjects (158 hypomethylated and 351 hypermethylated, see the “Methods” section). The majority (*N* = 281, 55%) of DMRs had an absolute mean methylation level difference of at least 5% (details in Additional file 5: Table S2).

**Fig. 2:**
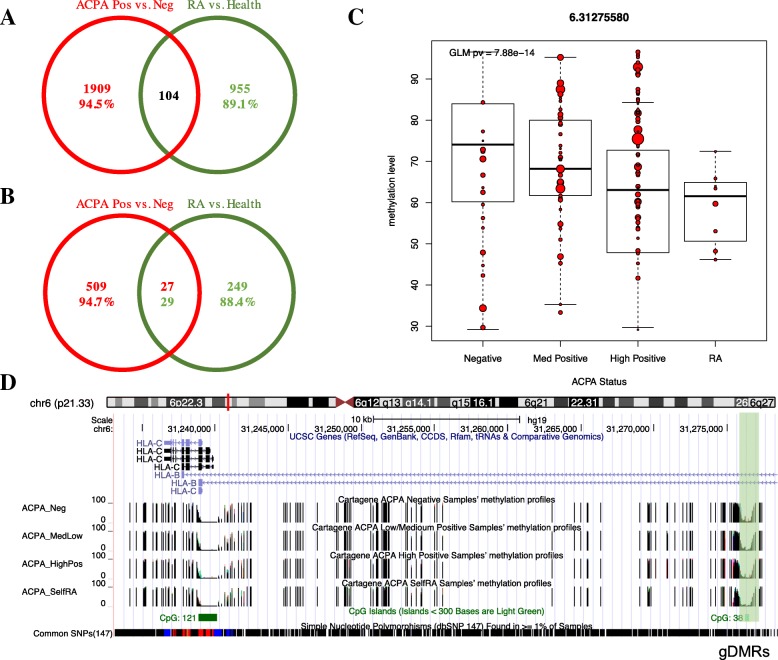
EWAS analysis in ACPA-measured subjects, self-reported RA patients, and dose-dependent DNA methylation analysis. **a** Venn diagram of DMCs inferred from ACPA-positive vs. ACPA-negative and self-reported RA vs. non-RA healthy. **b** Venn diagram of DMRs inferred from ACPA-positive vs. ACPA-negative and self-reported RA vs. non-RA healthy. **c** Three groups were analyzed in an ordinal model relating ACPA level to methylation (ACPA-negative, ACPA-medium-positive, and ACPA-high-positive). A CpG site located at HLA region (e.g., chr6: 31275580) from the ACPA-associated DMRs shows a hypomethylated pattern where methylation decreases from left to right. Methylation level profiles of self-reported RA were also added but not included in the three-group analyses. Dot size represents the sequencing read coverage of the given CpG site per sample. **d** USCS track browser for the corresponded DMR region associated with the site in **c**

We then compared DNA methylation differences between the 8 subjects with self-reported RA and all other sampled CARTaGENE subjects with measured ACPA (positive or negative). In models including blood cell-type adjustments (model III), there were 955 DMCs (435 hypomethylated and 520 hypermethylated) (Additional file 5: Table S3). These DMCs could be grouped into 249 DMRs (81 hypomethylated and 168 hypermethylated). Comparing with the identified DMCs from the comparison of ACPA-positive vs. ACPA-negative subjects, only 104 DMCs were shared; 94.5% of ACPA-positive vs. ACPA-negative DMCs and 89.1% of self-reported RA vs. healthy DMCs were specific (Fig. 2a). Similarly, only 27 DMRs out of 509 ACPA-positive vs. ACPA-negative DMRs and 29 out of 249 self-reported RA vs. healthy DMRs were shared (Fig. 2b).

### Dose-dependent DNA methylation

Considering the results of models II and III and the suggestion of trends in methylation levels, our next analysis explored “dose effects” of DNA methylation on ACPA status. The ACPA variable was grouped into three categories: negative, low-/medium-positive, and high-positive, and fit an ordinal model (see the “Methods” section) called “dose-effect model” hereinafter (model IV in Table 2). This resulted in 4475 DMCs (termed as ACPA-associated DMCs) and 1303 DMRs (termed as ACPA-associated DMRs). These DMRs included 455 genes and 315 intergenic regions (Additional file 5: Table S4). Next, the ACPA-associated DMCs were filtered to identify those where the methylation changes demonstrated an ordinal pattern (i.e., a directional effect) across the ACPA levels, and then further filtered to find DMCs where the RA patients demonstrated the same directional trend. In this manner, 869 hypodirectional DMCs and 761 hyperdirectional DMCs were defined from the ordinal model, together with 62 hypodirectional and 60 hyperdirectional DMRs. Among these sites, RA patients showed methylation levels that followed the same direction of change for 134 hypodirectional and 103 hyperdirectional DMCs (Additional file 5: Table S5). Moreover, 12 hypo and 6 hyperdirectional DMRs were identified in a similar manner. For example, a CpG located at chr6:31275580 (upstream of HLA-C region) is one of the top hypomethylated CpGs showing a directionally consistent decrease in DNA methylation levels (> 10%) when comparing ACPA-negative individuals, medium-positive individuals, high-positive individuals, and then RA patients (Fig. 2c). Furthermore, four DMCs around this site formed a DMR region (Fig. 2d).

**Fig. 3:**
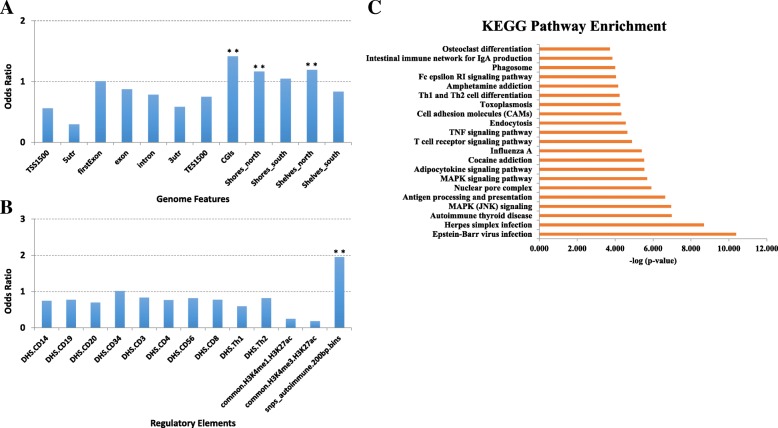
Results of genomic element enrichment analysis of ACPA-associated DMRs. **a** Genome feature enrichment analysis. **b** Regulatory element enrichment analysis. **c** KEGG pathway enrichment analysis for DMRs. **p value < 0.001

We then explored the enrichment of ACPA-associated DMRs in different genomic contexts. There were fewer of these associated DMR TSS regions and UTR regions than would be expected (e.g., fold change, FC = 0.56, 0.3, and 0.58 in TSS 1500 bp, 5’ UTR and 3’ UTR regions, respectively). However, ACPA-associated DMRs showed enrichment in CGI-associated regions, especially for the CGIs and CGI-shore regions (up to FC = 1.4 reaching enrichment *p* value = 1.2e−154) (Fig. 3a). Meanwhile, we did not see any enrichment of these DMRs in various regulatory elements including DNA accessibility regions and histone modification peak regions (Fig. 3b). However, interestingly, these DMRs were highly enriched in the auto-immune SNP-associated regions (FC = 1.96, *p* value = 4e−4). These auto-immune SNP-associated regions are defined as 200 bp up- and downstream from SNPs identified from genome-wide association studies of auto-immune disease, as well as SNPs in linkage disequilibrium with these key SNPs with *r*^2^ > 0.8 [31]. In pathway enrichment analyses of genes in the vicinity of significant CpGs, there was enrichment of several viral infection (Epstein-Barr virus, EBV, herpes simplex, influenza A), MAPK signaling, T cell activation, and osteoclast differentiation pathways (Fig. 3c).

### meQTLs and ACPA-associated DMRs

Given the substantial enrichment of ACPA-associated DMRs in auto-immune SNP-associated regions, we examined the overlap between these DMRs and previously reported RA-associated SNPs from GWAS analyses [32]. In 74 (5.7%) of the 1303 ACPA-associated DMRs found with model IV, there was an RA-associated SNP within 500 kilobases (kb). For instance, one DMR on chr22 (chr22:39747459-39747832) aligns with SNP rs909685 (associated with SYNGR1), which is located within the DMR. When looking only at the 122 directional ACPA-associated DMRs, RA-SNP associations had been previously observed for 9 regions (7.4%). Hence, genotypes may be underlying confounders for some of our identified ACPA-methylation associations. We therefore performed local meQTLs analyses and then aligned the associated DMCs/DMRs to the identified meQTLs. Briefly, we tested for associations at 2,258,466 SNP-CpG pairs located no more than 250 K from each other: this analysis included 1,001,116 CpGs and 67,399 SNPs. Among these tests, 61,260 CpG-SNP pairs showed significance (at *p* value < 5e−8, covering 22,657 independent CpGs and 11,069 unique SNPs). The 11,069 SNPs form the genome-wide significant set of meQTLs for further analyses.

Of the 4475 ACPA-associated DMCs identified through ordinal model analyses in model IV, there were 3127 CpGs where an appropriate SNP was available that was therefore included in the meQTL analysis above. Accordingly, 1068 of 1303 ACPA-associated DMRs have testable genetic influences. Reanalysis of the ACPA-methylation associations for these 3127 DMCs and those involved in 1068 DMRs was therefore undertaken including the identified lead meQTL SNP for the CpG in the model. After adjustment for lead meQTL genotype, many of the ACPA-methylation associations lost strength; however, 381 DMCs and 227 DMRs remained statistically significant (at Bonferroni multiple testing *q* value < 0.01). Therefore, we further categorized the 1068 ACPA-associated DMRs into two groups, genetically influenced DMRs (gDMRs) and non-genetically influenced DMRs (ngDMRs), based on whether or not they remained significant after genotype adjustment. That is, there were 841 (78.7%) gDMRs and 227 (21.3%) ngDMRs. Extracted from the full list of gDMRs and ngDMRs in Additional file 5: Table S4, Table 3 lists the top 10 most significant DMRs with absolute mean methylation difference between ACPA high-positive and negative group ≥ 10%, separated as just described, with their associated genes.

**Table 3:** Results of top ACPC-associated DMRs separated into those where there was a SNP influencing methylation levels nearby (gDMRs) and those where a nearby associated SNP (ngDMRs) was not found

#chr	Start	End	#DMC	*q* values, mean	Methdiff, mean	Distance to TSS	Gene name
gDMRs							
chr10	43140840	43140878	4	4.68E−06	12	− 6574	ZNF33B
chr13	114579169	114579173	4	6.20E−06	15	7288	LINC00454
chr19	13874993	13875015	4	5.42E−05	− 12	− 324	MRI1
chr4	185189026	185189056	4	0.000174	24	− 49,927	ENPP6
chr12	42539166	42539215	8	6.22E−04	11	− 518	GXYLT1
chr12	124858272	124858284	4	1.03E−03	− 11	− 36,490	MIR6880
chr12	11699957	11700234	22	0.00154	− 14	− 868	LINC01252
chr6	26755655	26755778	6	1.98E−03	12	− 95,737	ZNF322
chr5	171203344	171203485	14	2.31E−03	11	− 9461	SMIM23
chr15	91474603	91474621	4	0.00294	− 10	1187	HDDC3
ngDMRs							
chr13	114579169	114579173	4	6.20E−06	15	7288	LINC00454
chr14	24944437	24944579	10	8.84E−05	− 12	32,349	LOC101927045
chr2	1370435	1370661	24	4.05E−04	11	− 46,685	TPO
chr12	42539166	42539215	8	6.22E−04	11	− 518	GXYLT1
chr20	29523738	29524187	27	1.42E−03	− 14	46,400	LINC01598
chr2	91850066	91850070	4	1.71E−03	− 16	− 2093	LOC654342
chr6	26755655	26755778	6	1.98E−03	12	− 95,737	ZNF322
chr7	93996387	93996403	6	2.69E−03	− 11	− 27,478	COL1A2
chr15	91474603	91474621	4	0.00294	− 10	1187	HDDC3
chr14	106865983	106866072	4	3.05E−03	− 10	− 72,417	LINC00221

### Replication in an independent RA cohort

To further validate the MCC-Seq-based EWAS analysis, methylomes of CD4+ T cells from 9 RA cases and 13 healthy controls without RA were sequenced using the same panel. Here, similar binomial regression model (model V), but without additional covariates due to the small sample size, was applied. Results from this analysis were compared to results from the CARTaGENE discovery cohort. In this RA cohort, 3,262,817 CpGs had sufficient coverage to be tested. At a *p* value < 0.05, 116 (15.6%) of 743 DMCs also tested in the CARTaGENE data showed evidence of replication, as defined by the same direction in DNA methylation change. Compared to the DMCs set (*n* = 321,036) from tested CpGs in the RA cohort, and showing EWAS *q* value < 0.05, 12.2-fold enrichment (with *p* value = 4.9e−14) of overlapping significant DMCs was observed. Even at nominal significance cutoff for smaller RA cohort, significant enrichment for DMCs with overlapping associations was observed (fold change of 1.5 and *p* value ≤ 1.2e−5) (Additional file 5: Table S6). After adjusting these 116 DMCs by cis-genotype information, the strength of association was reduced (to non-significant) in 36 (30%), indicating that genetic effects might be inducing some of the methylation-phenotype associations in both datasets.

Finally, replication of results between this validation cohort and our analysis of self-reported RA in the CARTaGENE subjects was assessed. Of the 249 DMRs observed in the latter (model III, Table 2), 208 regions also met the criteria for analysis in the validation T cell cohort. Among these 208 common tested regions, we were able to replicate 38% by finding statistical significance at overlapping DMCs with a nominal *p* value < 0.05. These 80 DMRs are listed in Additional file 5: Table S7.

## Discussion

This is the first genome-wide sequencing-based DNA methylation association analysis of ACPA-positive and ACPA-negative subjects. Distinct DNA methylation loci are present in ACPA-positive vs. ACPA-negative individuals and in RA vs. ACPA-positive subjects without RA. Common directional loci were found across ACPA-negative, ACPA-low/medium-positive, ACPA-high-positive, and RA patients. The findings of altered DNA methylation in ACPA-positive subjects without RA and the directional methylation changes support the existence of possible causal pathways between epigenetic abnormalities and RA.

We rigorously adjusted the EWAS models with measured blood cell proportions using known differential counts and observed that the majority of significant signals were maintained (e.g., 85% of the ACPA-negative vs. ACPA-positive DMCs remained significant after blood composition correction). In fact, blood cell proportion corrections are essential for EWAS studies using whole blood samples [33]. If measured blood cell proportions are not available, computational approaches are available to deconvolute the compositions and include those as covariates in models [34–39].

The high-density results highlighted genes showing ACPA-associated DMRs involved in several viral infections, with the most significant being the EBV infection pathway. EBV has previously been suggested to be implicated in the etiology of RA [40]. A meta-analysis study showed the risk of RA after EBV infection [41]. More recently, Harley et al. suggested that EBNA2, the product of EBV, directly modulates gene expression in autoimmune disease loci [42]. This DNA methylation analysis thus provides support for the hypothesis that environmental triggers including EBV contribute to the pathophysiology of RA prior to the onset of clinical disease. This is consistent with the “multi-hit” theory [43, 44] whereby complex autoimmune diseases like RA arise as a consequence of underlying risk factors (that may be genetic), but which lead to disease only in the presence of other events, such as viral infections. We hypothesized that altered DNA methylation prior to disease onset may be particularly relevant for environmental triggers. Consistent with this hypothesis, we observed methylation changes already present in ACPA-positive subjects, prior to the onset of disease, that were enriched in regions and genes related to viral infections.

The genes involved in immune-related pathways include CARD11, CSF2, MAP3K7, NFATC1, PAK4, NFKBIA, MAPK9, IFNAR2, FCGR2A, and SOCS3. Interestingly, most of them except CSF2 and FCGR2A were not associated with reported RA GWAS loci (CSF2 and FCGR2A were reported to associate with RA GWAS loci rs657075 and rs72717009, respectively [32]). Nevertheless, our meQTLs results revealed DMRs related to CSF2, NFATC1, NFKBIA, and MAPK9 that contained DMCs affected by some genetic effects. These results suggest that these DMRs might be a consequence of long-range haplotype effects of genetic variants on methylation, which is consistent with the results from Liu et al. where they found five out of nine DMCs were mediated by genetic variants [6].

Of note, MAP3K7 (TAK1), which is associated with an ngDMR (co-localized at a low-methylated region [24] which typically implies an enhancer-like regulatory element), is a kinase known to activate MAPK8/JNK and MAP2K4/MKK4 and plays a role in the cell response to environmental stresses. It has recently been reported as a new therapeutic target in RA [45, 46]. CARD11, an essential adaptor protein that activates the nuclear factor (NF)-κB signaling pathway, is reported to be involved in the pathogenesis of RA and could also be a potential therapeutic target [47]. IFNAR2 is one of the type I interferon receptors, which are currently considered as key factors in the development and regulation of autoimmune diseases such as RA [48, 49]. SOCS3, a member of the family of cytokine signaling proteins, was reported to show increased gene expression level changes in RA patients compared with healthy participants [50] and was a key signaling molecule in bone cell-mediated inflammatory responses [51–53]. These results highlighted the potential of our new sequencing-based technique to detect RA-relevant targets. In addition, RA-associated DMRs were enriched in enhancer-like regions. Future work will include integrating HiC-Seq [54, 55] data to detect the physical intersections between enhancers and promoters to identify the regulated genes.

In the pairwise comparisons, around 15.6% of RA-specific DMCs and nearly 38% of RA-specific DMRs were well replicated in an independent data set of RA patients and controls, where CD4+ T cells were available. Among them, IRF9, showing hypomethylation in the RA patients, has been reported to activate the JAK-STAT signaling pathway, which further triggers the induction of type I interferon response genes (IRG). JAK inhibitors are approved for the treatment of RA [56]. Given the limited sample size of (self-reported) RA patients in our study, current replication rates provide promising results regarding identifying RA-specific DNA methylation signals.

In contrast to the earlier study by Gomez-Cabrero et al. [57] where monozygotic (MZ) twins were analyzed with the HM450K technology, and with a smaller number of subjects, we employed a platform designed on known regulatory elements in human circulating leukocytes. Our panel captured more than 4 million CpGs for analysis, which is roughly 10-fold larger than the HM450K and 6-fold larger than the Human Methylation EPIC (EPIC) Bead Chips (Illumina, CA, USA) [58], providing the potential for discovering novel signals at an unprecedented level of resolution. We observed that 64.1% of the ACPA-positive and ACPA-negative DMRs contain CpGs not found on the Illumina EPIC array probes (and this proportion rises to > 70.2% for the Infinium HM450K array probes). In addition, by comparing with Gomez-Cabrero et al.’s 18 candidate DMRs, none of them overlapped with our DMR list and only 5 of them replicated with at least one CpG showing nominal *p* value < 0.05 in our analysis. This low replicate rate might be due to the overall low overlap rate between our DMC set and the HM450K probes (e.g., when overlapping our hits for model IV with HM450K, only observed 7% of them overlapped). Meanwhile, these differences could also be due to sampling variations, differences in platforms/methods, and/or differences in the demographics of the population.

We observed around 79% of the ACPA-associated DMRs or RA-associated DMCs were influenced by cis-regulatory SNPs. These SNPs were extracted from the MCC-Seq data directly, once again showing the advantages of a sequencing-based technique over array-based platforms by generating CpG methylation and neighboring SNPs simultaneously. Consequently, our approach can address both genetically and environmentally mediated DNA methylation changes related to the disease.

## Conclusions

This is the first bisulfite sequencing-based EWAS study in autoimmune disease using a population-based blood sampling for individuals at elevated risk for RA. After controlling for known confounding of blood cell sub-types in EWAS, this study uncovered both genetically mediated and putatively environmentally induced signals functionally linking viral infections to ACPA positivity. This data also supports the hypothesis of a causal link between epigenetic abnormalities and RA.

## Additional files

Additional file 1: **Figure S1.** Regions with at least 3 CpGs fulfilling these criteria were considered differentially methylated regions. (DOCX 44 kb)

Additional file 2: **Figure S2.** Details on read and sample coverage. (PDF 140 kb)

Additional file 3: **Figure S3.** At a *q* value < 0.1 (from model II), 85.3% of the DMCs in model I remained significant in model II. (PDF 148 kb)

Additional file 4: **Tables S1–S7.** This file contains Tables S1–S7. (PDF 16 kb)

Additional file 5: **Table S1.** Results of DMCs from ACPA Positive vs. Negative comparison after blood composition adjustment. (XLSX 408T1 kb)
