# Supplementary material for: Rheumatoid arthritis-relevant DNA methylation changes identified in ACPA-positive asymptomatic individuals using methylome capture sequencing
Source: Clin Epigenetics. 2019 Jul 31;11:110. doi: 10.1186/s13148-019-0699-9 (PMC6668183; doi:10.1186/s13148-019-0699-9)
Supplement: Supplementary file 1 — Figure S1. Regions with at least 3 CpGs fulfilling these criteria were considered differentially methylated regions. (DOCX 44 kb) [file 13148_2019_699_MOESM1_ESM.docx]

**Rheumatoid arthritis-relevant DNA methylation changes identified in ACPA-positive asymptomatic individuals using methylome capture sequencing**

Xiaojian Shao^1,2,11^, Marie Hudson^3,4,5^, Ines Colmegna^3,6^, Celia MT Greenwood^1,3,5^, Marvin J Fritzler^7^, Philip Awadalla^8,9^, Tomi Pastinen*^1,10^, Sasha Bernatsky*^3,6^

*equal roles as senior author

^1^ Department of Human Genetics, McGill University, Montréal, Canada.

^2^ The McGill University and Génome Québec Innovation Centre, Montréal, Canada.

^3^ Department of Medicine, McGill University, Montréal, Canada.

^4^ Division of Rheumatology, Jewish General Hospital, Montréal, Canada

^5^ Lady Davis Institute, Jewish General Hospital, Montréal, Canada

^6^ Division of Rheumatology, McGill University, Montréal, Canada

^7^ Cumming School of Medicine, University of Calgary, Calgary, Canada.

^8^ Ontario Institute for Cancer Research, Toronto, Canada

^9^ Department of Molecular Genetics, University of Toronto, Toronto, Canada

^10^Center for Pediatric Genomic Medicine, Children’s Mercy, Kansas City, MO, USA

^11^ Current address: Digital Technologies Research Centre, National Research Council Canada, Ottawa, Ontario, Canada

**CORRESPONDING AUTHOR:**

Sasha Bernatsky, Department of Medicine, McGill University, Montréal, Canada sasha.bernatsky@mcgill.ca

**Supplementary Information**

## Methylation sequencing protocol

The methylC-capture sequencing (MCC-Seq)[^1^](#_ENREF_1) [^2^](#_ENREF_2) protocol was developed and optimized in Roche NimbleGen R&D where SeqCap Epi Enrichment System protocol (Roche NimbleGen) was carried out for the capture. Specifically, a whole-genome sequencing library was prepared and bisulfite converted, amplified and a capture enriching for targeted bisulfite-converted DNA fragments was carried out. The captured DNA is further amplified and sequenced. Sequencing of the MCC-Seq libraries was performed on the Illumina HiSeq2000/2500 system using 100 bp paired-end sequencing. More specifically, whole-genome sequencing libraries were generated from 700 to 1,000 ng of genomic DNA spiked with 0.1% (w/w) unmethylated λ DNA (Promega) previously fragmented to 300–400 bp peak sizes using the Covaris focused-ultrasonicator E210. Fragment size was controlled on a Bioanalyzer DNA 1000 Chip (Agilent) and the KAPA High Throughput Library Preparation Kit (KAPA Biosystems) was applied. End repair of the generated dsDNA with 3′- or 5′-overhangs, adenylation of 3′-ends, adaptor ligation and clean-up steps were carried out as per KAPA Biosystems' recommendations. The cleaned-up ligation product was then analysed on a Bioanalyzer High Sensitivity DNA Chip (Agilent) and quantified by PicoGreen (Life Technologies). Samples were then bisulfite converted using the Epitect Fast DNA Bisulfite Kit (Qiagen), according to the manufacturer's protocol. Bisulfite-converted DNA was quantified using OliGreen (Life Technologies) and, based on quantity, amplified by 9–12 cycles of PCR using the Kapa Hifi Uracil+DNA polymerase (KAPA Biosystems), according to the manufacturer's protocol. The amplified libraries were purified using Ampure Beads and validated on Bioanalyzer High Sensitivity DNA Chips, and quantified by PicoGreen.

The hybridization procedure of the amplified bisulfite-converted library was performed as described by the manufacturer, using 1 μg of total input of library, which was evenly divided by the libraries to be multiplexed, and incubated at 47 °C for 72 h. Washing and recovering of the captured library, as well as PCR amplification and final purification, were carried out as recommended by the manufacturer. Quality, concentration and size distribution of the captured library was determined by Bioanalyzer High Sensitivity DNA Chips.

## MCC-Seq data processing

MCC-Seq paired-end raw reads were first trimmed for quality (phred33 >= 30), length (n >= 50) and Illumina adapters using Trimmomatic v. 0.36 ^[3](#_ENREF_3)^. The survival reads were then aligned per sequencing lane to the pre-indexed reference genome by Bismark software v.0.18.2 [^4^](#_ENREF_4) with bowtie2 v. 2.3.1 [^5^](#_ENREF_5) in pair-end mode and with default parameters. Lane bam files were merged and then de-duplicated using picard v. 2.9.0 [^6^](#_ENREF_6). Methylation calls were obtained using the Bismark “bismark_methylation_extractor”. BisSNP v0.82.2 [^7^](#_ENREF_7) was run on the de-duplicated bam files to call variants. To avoid potential biases in downstream analyses, CpGs overlapping a SNP (from dbSNPs 137) and CpGs located within ENCODE DAC blacklisted regions or Duke excluded regions (<http://hgwdev.cse.ucsc.edu/cgi-bin/hgFileUi?db=hg19&g=wgEncodeMapability>) were discarded [^8^](#_ENREF_8). CpGs covered by less than five reads were also discarded.

**Reference**

1. Allum F, Shao XJ, Guenard F, et al. Characterization of functional methylomes by next-generation capture sequencing identifies novel disease-associated variants. *Nature communications* 2015;6 doi: 10.1038/Ncomms8211

2. Cheung WA, Shao XJ, Morin A, et al. Functional variation in allelic methylomes underscores a strong genetic contribution and reveals novel epigenetic alterations in the human epigenome. *Genome biology* 2017;18 doi: 10.1186/S13059-017-1173-7

3. Bolger AM, Lohse M, Usadel B. Trimmomatic: a flexible trimmer for Illumina sequence data. *Bioinformatics* 2014;30(15):2114-20. doi: 10.1093/bioinformatics/btu170

4. Krueger F, Andrews SR. Bismark: a flexible aligner and methylation caller for Bisulfite-Seq applications. *Bioinformatics* 2011;27(11):1571-2. doi: 10.1093/bioinformatics/btr167

5. Langmead B, Salzberg SL. Fast gapped-read alignment with Bowtie 2. *Nature methods* 2012;9(4):357-9. doi: 10.1038/nmeth.1923

6. <http://broadinstitute.github.io/picard/>.

7. Liu Y, Siegmund KD, Laird PW, et al. Bis-SNP: combined DNA methylation and SNP calling for Bisulfite-seq data. *Genome biology* 2012;13(7):R61. doi: 10.1186/gb-2012-13-7-r61

8. Consortium EP. An integrated encyclopedia of DNA elements in the human genome. *Nature* 2012;489(7414):57-74. doi: 10.1038/nature11247
