# Supplementary material for: Rheumatoid arthritis-relevant DNA methylation changes identified in ACPA-positive asymptomatic individuals using methylome capture sequencing
Source: Clin Epigenetics. 2019 Jul 31;11:110. doi: 10.1186/s13148-019-0699-9 (PMC6668183; doi:10.1186/s13148-019-0699-9)
Supplement: Supplementary file 2 — Figure S2. Details on read and sample coverage. (PDF 140 kb) [file 13148_2019_699_MOESM2_ESM.pdf]

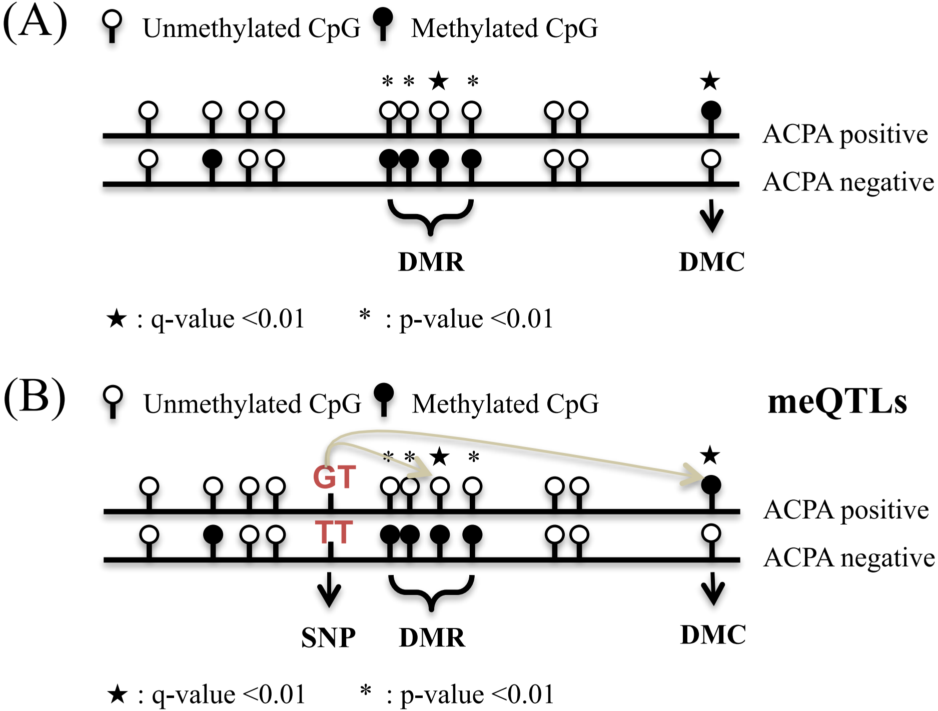
(A)
Unmethylated CpG
Methylated CpG
ACPA positive
ACPA negative
DMR
DMC
★ : q-value <0.01
* : p-value <0.01
(B)
Unmethylated CpG
Methylated CpG
meQTLs
GT
TT
ACPA positive
ACPA negative
SNP
DMR
DMC
★ : q-value <0.01
* : p-value <0.01
