# Supplementary figures and images for: Rheumatoid arthritis-relevant DNA methylation changes identified in ACPA-positive asymptomatic individuals using methylome capture sequencing
Source: Clin Epigenetics. 2019 Jul 31;11:110. doi: 10.1186/s13148-019-0699-9 (PMC6668183; doi:10.1186/s13148-019-0699-9)

**CpG methylation level distribution**

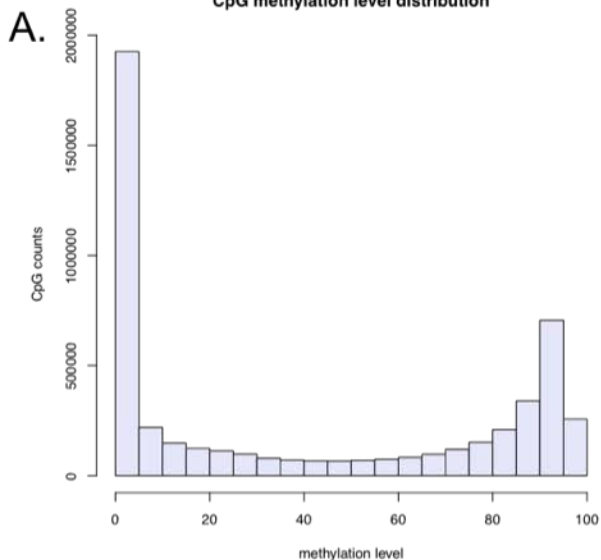

**CpG read coverage distribution**

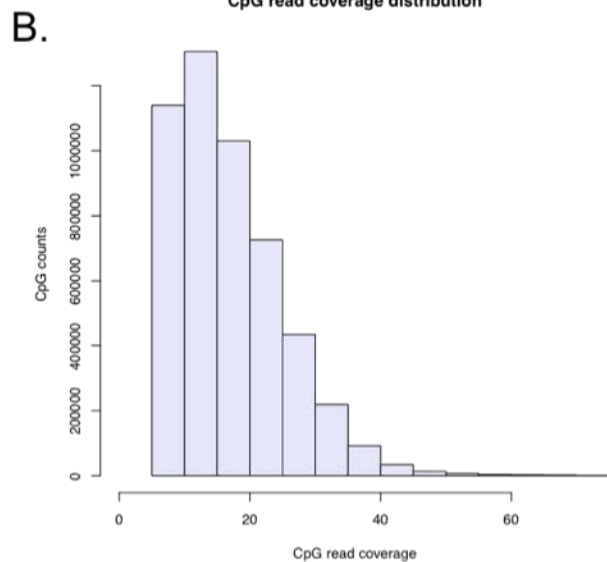

**Sample coverage per CpG**

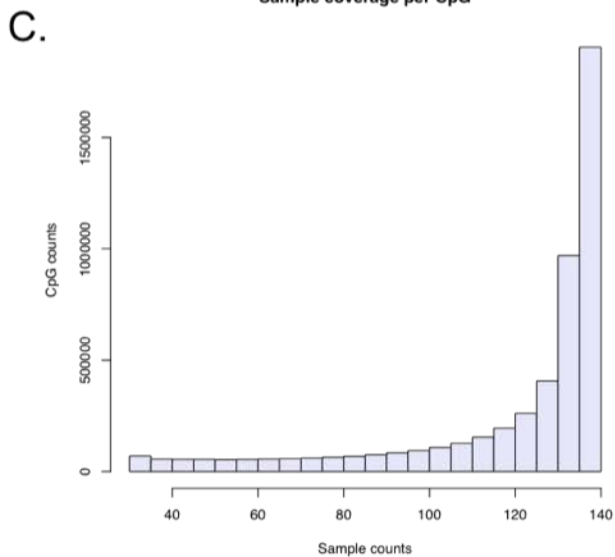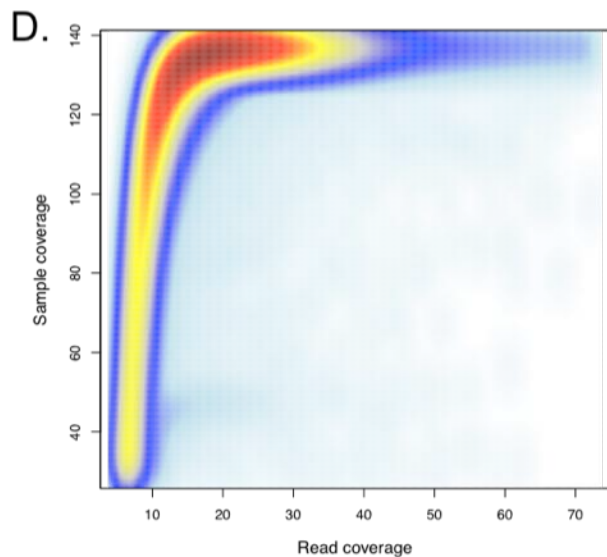

Supplement: Supplementary file 3 — Figure S3. At a q value < 0.1 (from model II), 85.3% of the DMCs in model I remained significant in model II. (PDF 148 kb) [file 13148_2019_699_MOESM3_ESM.pdf]

# q-value correlation between different DMC sets

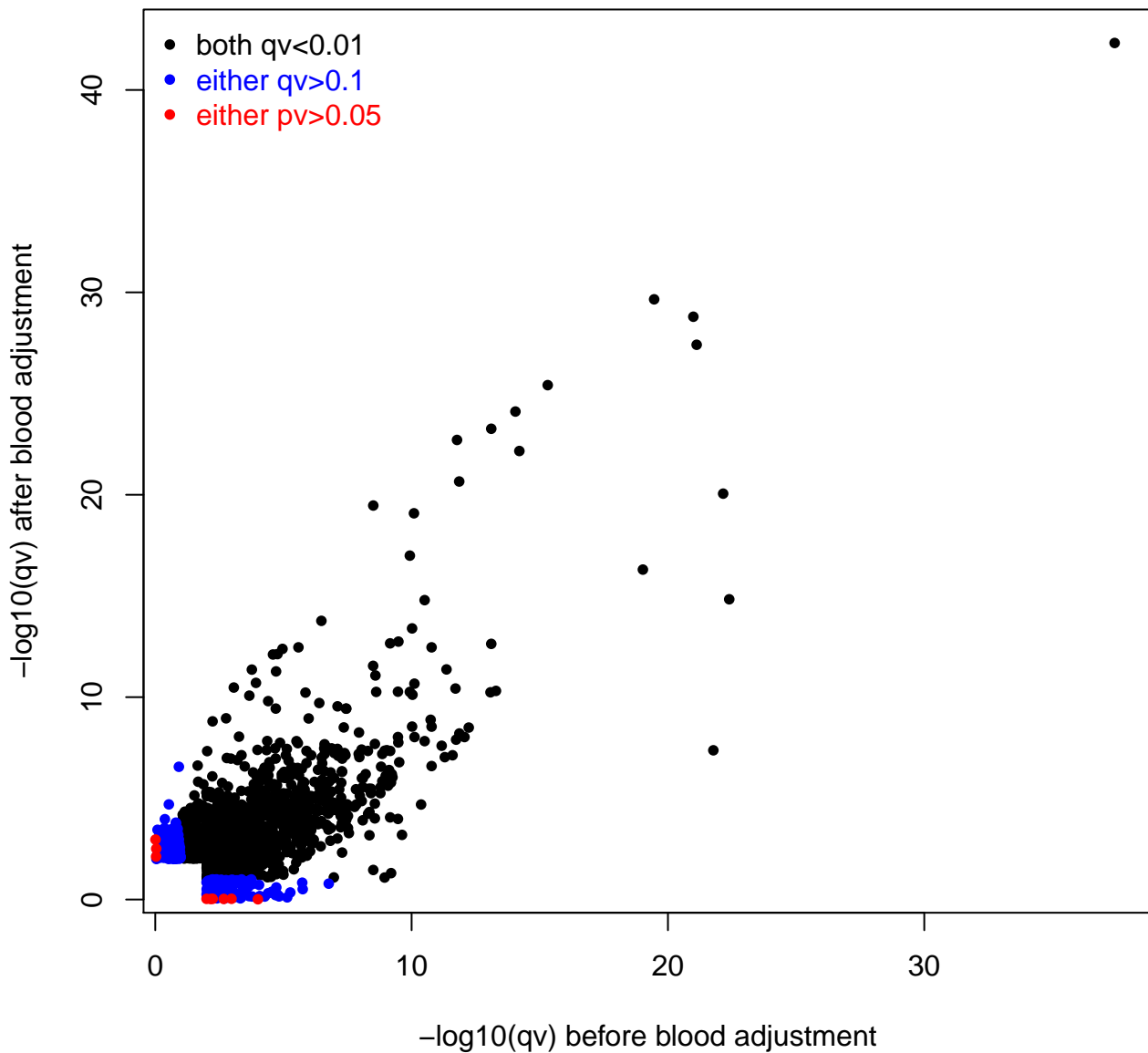

Supplement: Supplementary file 4 — Tables S1–S7. This file contains Tables S1–S7. (PDF 16 kb) [file 13148_2019_699_MOESM4_ESM.pdf]
